# A new model to describe small-angle neutron scattering from foams

**DOI:** 10.1107/S1600576722004691

**Published:** 2022-06-23

**Authors:** Matthias Kühnhammer, Larissa Braun, Michael Ludwig, Olaf Soltwedel, Leonardo Chiappisi, Regine von Klitzing

**Affiliations:** a Institut für Physik Kondensierter Materie, Technische Universität Darmstadt, Hochschulstraße 8, 64289 Darmstadt, Germany; b Institut Laue–Langevin, 71 avenue des Martyrs, F-38042 Grenoble, France; Oak Ridge National Laboratory, USA, and North Carolina State University, USA

**Keywords:** foams, foam films, small-angle neutron scattering, neutron reflectometry, thin-film pressure balances

## Abstract

The modelling of scattering data from foams is very challenging due to the complex structure of foams and is therefore often reduced to the fitting of single peak positions or feature mimicking. This article presents a more elaborate model to describe the small-angle neutron scattering (SANS) data from foams. The model takes into account the geometry of the foam bubbles and is based on an incoherent superposition of the reflectivity curves arising from the foam films and the small-angle scattering (SAS) contribution from the plateau borders. The model is capable of describing the complete scattering curve of a foam stabilized by the standard cationic surfactant tetradecyltrimethylammonium bromide (C_14_TAB) with different water contents, *i.e.* different drainage states, and provides information on the thickness distribution of liquid films inside the foam. The mean film thickness decreases with decreasing water content because of drainage, from 28 to 22 nm, while the polydispersity increases. These results are in good agreement with the film thicknesses of individual horizontal foam films studied with a thin-film pressure balance.

## Abstract 2

A new model for the interpretation of small-angle neutron scattering data from aqueous foams is presented and validated using experimental data from a model foam system.

## 1. Introduction

Liquid foams are a dispersion of gas in a continuous liquid phase, and they have many applications in everyday life and industrial processes (Prud’homme & Khan, 1995 ▸; Schramm, 2005 ▸; Exerowa *et al.*, 2019 ▸). The continuous liquid phase is often considered as a hierarchical network with structures of different sizes. The smallest building blocks of this network are the foam films, which have a thickness of several tens of nanometres. These are the liquid films which separate two bubbles. The connections between three foam films are called plateau borders, and these typically have thicknesses of several tens of micrometres (Koehler *et al.*, 2002 ▸, 2004 ▸). Their length is coupled to the foam bubble diameter, which ranges from several micrometres to centimetres, depending on the drainage state and foaming technique (Drenckhan & Saint-Jalmes, 2015 ▸). The intersection of plateau borders is called a node (Weaire & Hutzler, 1999 ▸; Koehler *et al.*, 2000 ▸).

A liquid foam is an intrinsically unstable system and its architecture changes over time. The main mechanisms are coarsening (diffusion of gas between bubbles) (Briceño-Ahumada & Langevin, 2017 ▸), gravitational drainage (Kruglyakov *et al.*, 2008 ▸) and coalescence (merging of bubbles by rupturing of films) (Rio & Biance, 2014 ▸).

This complex and evolving structure and the large difference in the refractive indices of the gas and (aqueous) liquid phases limits light microscopy to very dry (*i.e.* low liquid volume fraction) foams with large bubbles (Monnereau & Vignes-Adler, 1998 ▸; Fetterman *et al.*, 2000 ▸). A more powerful technique for the investigation of the three-dimensional structure of foams is X-ray tomography, which allows a detailed analysis of bubble size and shape inside the foam (Lambert *et al.*, 2005 ▸; Meagher *et al.*, 2011 ▸). However, a determination of film thickness inside the foam is not possible with this method. Investigations regarding foam films have for a long time been limited to experiments with single horizontal foam films in a pressurized chamber and interferometric analysis of the thickness. The initial experiments were performed by Scheludko and Exerowa with what is now known as the Scheludko cell, which consists of a glass ring in which a liquid film is generated (Scheludko, 1957 ▸; Scheludko & Exerowa, 1959 ▸, 1960 ▸). Numerous studies were performed with similar experimental setups, referred to as thin-film pressure balances (TFPBs), with a variety of systems (Bergeron, 1997 ▸, 1999 ▸; Stubenrauch *et al.*, 2002 ▸; Stubenrauch & Klitzing, 2003 ▸; Schulze-Schlarmann *et al.*, 2006 ▸; Schelero *et al.*, 2014 ▸; Uhlig *et al.*, 2016 ▸, 2020 ▸). Despite the valuable insight these studies provided regarding the forces between liquid films, the results are often not consistent with macroscopic foam properties like foam stability, probably because a single horizontal liquid film is an oversimplification of the complex structure of foams (Braun *et al.*, 2020 ▸).

Axelos & Boué (2003 ▸) were the first to use small-angle neutron scattering (SANS) to study liquid foams. SANS is an excellent tool to probe liquid foams. On the one hand, air and D_2_O provide excellent neutron scattering length density (SLD) contrast conditions, with the possibility of matching the neutron SLD of the surfactant by mixing D_2_O and H_2_O. On the other hand, the large neutron beam (tens of millimetres) allows the simultaneous probing of a large number of bubbles, providing a representative ensemble equivalent. Moreover, the probed *q* range [*q* = (4π/λ)sinθ, where θ is half the scattering angle and λ is the wavelength of the incident radiation] also allows access to structural features in the range 1–100 nm, which are hard to access using other techniques.

Axelos and Boué investigated foams stabilized by sodium dodecyl sulfate (SDS) at various concentrations above its critical micelle concentration (CMC). In wet foams they observed similar scattering features to those in the corresponding micellar bulk solution and concluded the presence of micelles inside the foam. Upon drainage another peak was observed in the scattering curves, which they interpreted as interference between the two gas/liquid interfaces of the foam film. This allowed the determination of the film thickness inside the foam, which was 16–18 nm depending on the SDS concentration. Subsequent publications interpreted the oscillations in the scattering signal attributed to the film thickness in terms of reflectometry from randomly oriented mirrors (Ropers *et al.*, 2008 ▸; Schmidt *et al.*, 2010 ▸). One of the justifications for this interpretation was that the first maximum of these oscillations appeared at a value of the scattering vector *q* close to the critical edge *q*_c_ of a hypothetical reflectivity experiment with a single liquid layer in air and did not change during drainage. Following this interpretation, the film thickness *d* can be extracted from the period of the oscillations Δ*q* via *d* = 2π/Δ*q*. This interpretation was also used by Micheau *et al.* (2013 ▸), studying foams stabilized by nonaoxyethylene oleylether carboxylic acid. Yada *et al.* (2020 ▸) followed the interpretation of Axelos and Boué and extracted foam film thicknesses from a single peak position. Recently, Perticaroli *et al.* (2020 ▸) and Hohenschutz *et al.* (2021 ▸) used the reflectivity-based interpretation and modelled a reflectivity curve of a single liquid layer of thickness *d* to match the oscillations observed in their data. Hurcom *et al.* (2014 ▸) and Mansour *et al.* (2017 ▸, 2019 ▸) followed a different interpretation and were the first to model the full scattering curves of foams. They studied foams stabilized by polymeric and small-molecule surfactants and polymer/surfactant mixtures. They attributed the oscillations to (surface-induced) lamellar structures inside the foam films and fitted the data with a paracrystalline model, which yielded micrometre-sized film thicknesses in wet foams.

Given the complex structure of foams, several scattering and reflectivity processes might occur, as pointed out by Mikhailovskaya *et al.* (2017 ▸). Depending on the drainage state of the foam and the stabilizer used, different processes might dominate, which makes a unified description of these scattering curves very challenging. Most articles following the reflectivity-based interpretation mention that the complete scattering curve is a combination of Porod scattering (*I* ∝ *Bq* ^−4^) and a reflectivity contribution (Ropers *et al.*, 2008 ▸; Micheau *et al.*, 2013 ▸; Perticaroli *et al.*, 2020 ▸; Hohenschutz *et al.*, 2021 ▸). However, in these papers no attempt at modelling the full scattering curve was made.

In this article a new approach to the modelling of SANS curves from foams is presented. The model uses an incoherent superposition of a multitude of individual reflectivity curves and therefore explicitly accounts for the polydispersity in film thickness in the foam and a small-angle scattering (SAS) contribution. Furthermore, the model takes into account the spherical shape of foam bubbles in wet foams. In this way the complete scattering curve of a foam stabilized by the standard surfactant tetradecyltrimethylammonium bromide (C_14_TAB) can be described. It allows the extraction of detailed information about the evolution and distribution of film thicknesses and thus improves the understanding of the internal structure of foams.

## 2. Experimental

### 2.1. Materials

C_14_TAB (≥98%) was purchased from Sigma–Aldrich (St Louis, Missouri, USA) and recrystallized three times in acetone with traces of ethanol. D_2_O (99.9% D) was purchased from Eurisotop (Saclay, France) and was used as received. Deionized H_2_O with a specific resistance of 18.2 MΩ cm was obtained from a MilliQ water purification system (Merck KGaA, Darmstadt, Germany).

### 2.2. Thin-film pressure balance

Disjoining pressure isotherms of individual horizontal foam films of a C_14_TAB solution (*c* = CMC = 3.5 m*M*) were measured with a TFPB using the porous-plate technique (Mysels & Jones, 1966 ▸; Scheludko & Exerowa, 1960 ▸). In our custom-built setup, the film was formed in a 1 mm hole drilled into a porous glass disc [pore size 10–16 µm, porosity P16 (ISO Standard No. 4793; ISO, 1980 ▸)]. The film holder was placed in a sealed stainless steel chamber, which also contained a reservoir with surfactant solution to ensure a saturated atmosphere, thereby preventing film drainage. Before each measurement, the film holder was immersed in the surfactant solution for at least 2 h to equilibrate the porous glass disc with the solution. Disjoining pressure isotherms Π(*d*) were recorded by interferometrically measuring the equilibrium film thickness *d* as function of pressure inside the sample cell (Scheludko, 1967 ▸). The equilibrium film thickness was assumed to be reached once the intensity of the reflected light was constant for 20 min. The disjoining pressure isotherms presented in this article are each an average of five individual measurements.

### 2.3. Small-angle neutron scattering

SANS experiments were carried out on the D33 instrument at the Institut Laue–Langevin (Dewhurst *et al.*, 2016 ▸; Kühnhammer *et al.*, 2020 ▸). SANS measurements were performed with a circular neutron beam (diameter 15 mm) and a data acquisition time of 15 min at sample-to-detector distances of 1.7 m (front detector) and 10 m (rear detector) and a neutron wavelength of 0.46 nm, covering a *q* range of ∼0.05–4 nm^−1^. The collimation was set to 10.3 m. Processing and radial averaging of the 2D detector images was done with the *GRASP* software (https://www.ill.eu/users/support-labs-infrastructure/software-scientific-tools/grasp). Absolute units were obtained by normalizing for the direct beam. A custom-built sample cell, specifically designed for studying macroscopic foams with SANS, was used. Technical details are given in a previous publication (Kühnhammer *et al.*, 2021 ▸). Foams were produced from a C_14_TAB solution in D_2_O (*c* = 3.5 m*M*) by bubbling nitrogen gas at a rate of 10 ml min^−1^ through a porous glass plate [pore size 10–16 µm, porosity P16 (ISO 4793)] at the bottom of the cell. Once the foam level reached the top of the column, the flow rate was reduced to 1 ml min^−1^, resulting in a constant foam height. Once this steady state was reached, measurements at three different foam heights (*h* = 7, 12 and 16.5 cm above the foaming solution) were performed.

### 2.4. Model for the description of SANS curves

SANS curves were modelled with a purpose-written program using the Python programming language (Van Rossum & Drake, 2009 ▸) with the *SciPy* (Virtanen *et al.*, 2020 ▸), *NumPy* (Harris *et al.*, 2020 ▸) and *pandas* (McKinney, 2010 ▸; https://pandas.pydata.org/) packages. The model employs an incoherent superposition of a SAS decay and a reflectivity term. Incoherent superposition results in an additive description of the total scattering signal. This is justified, because the size of the foam bubbles (*r*_B_ ≃ 0.1–1 mm), and therefore the distance between two foam films, is larger than the coherence length of the neutrons.

The coherence length *l*_coh_ is estimated as

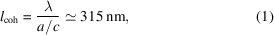

with the neutron wavelength λ = 0.46 nm, the aperture opening *a* = 15 mm and the collimation length *c* = 10.3 m.

The angular divergence ΔΘ in the SANS experiment is given by

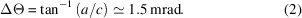

The maximum relative divergence of the scattering vector Δ*q*/*q* is 

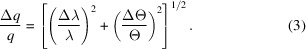

In monochromatic mode, the D33 instrument has a wavelength spread of Δλ/λ = 0.1 (Dewhurst *et al.*, 2016 ▸). At the position of the first feature in the scattering data (*q* ≃ 0.2 nm^−1^), Δ*q*/*q* is approximately 0.23, which is sufficient to resolve the feature.

The reflectivity term is a weighted sum of individual reflectivity curves of D_2_O layers in air with normally distributed thicknesses *d_i_*. This reflects the polydispersity of foam films in the macroscopic foam. This polydispersity can be interpreted either as the varying film thickness of a single foam film due to the curved bubble interface or as the thickness variation of all films in the foam. Since a single foam bubble is much larger than the coherence volume, the films (or film segments) within the coherence volume are approximated to have a monodisperse thickness.

Using a normal distribution, a weighting factor accounting for the polydispersity in film thickness *w_i_* is introduced, 

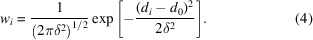

Here, *d*_0_ and δ are the mean and standard deviation, respectively, of the normally distributed film thicknesses and *d_i_* is the specific monodisperse film thickness weighted by *w_i_*. The region of interest of this distribution was set to be *d*_0_ ± 20 nm and the increment Δ*d_i_* = 0.1 nm. This results in 400 simulated reflectivity curves, which are summed to give the reflectivity contribution of the model.

In classic reflectometry experiments at flat substrates the angle of incidence is only governed by the experimental setup and does not change over the length of the sample. This is not the case for curved interfaces or many randomly oriented interfaces as in foams. In this case the projection of the incident beam on the surface of the scattering object has to be taken into account. This effect is similar to the footprint effect in reflectometry, where the projection of the incident beam under small angles becomes larger than the sample, leading to a reduction in the measured reflectivity. In the case of foams this means that foam films which are (nearly) parallel to the incident beam interact with fewer neutrons than foam films which enclose a larger angle with the incident beam. Therefore, the reflectivity contribution is modified by an angle correction *P*(Θ). Considering the many randomly oriented foam films probed by the neutron beam, *P*(Θ) is estimated by the projection of a parallel beam onto the surface of a sphere. The small plane-parallel foam films can also be seen as tangent planes to this sphere. Note that this sphere is only a theoretical construct and should not be interpreted as a foam bubble. For the calculation of *P*(Θ) two factors have to be considered: (i) the fraction of a sphere’s surface resulting in an angle of incidence Θ_i_ when illuminated with a parallel beam, and (ii) the probability of an angle of incidence Θ_i_ given by the projection of a parallel beam onto a circle.

The normalized fraction of a sphere’s surface resulting in an angle of incidence Θ_i_ is given by 

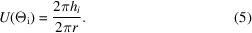

**Figure 1:**
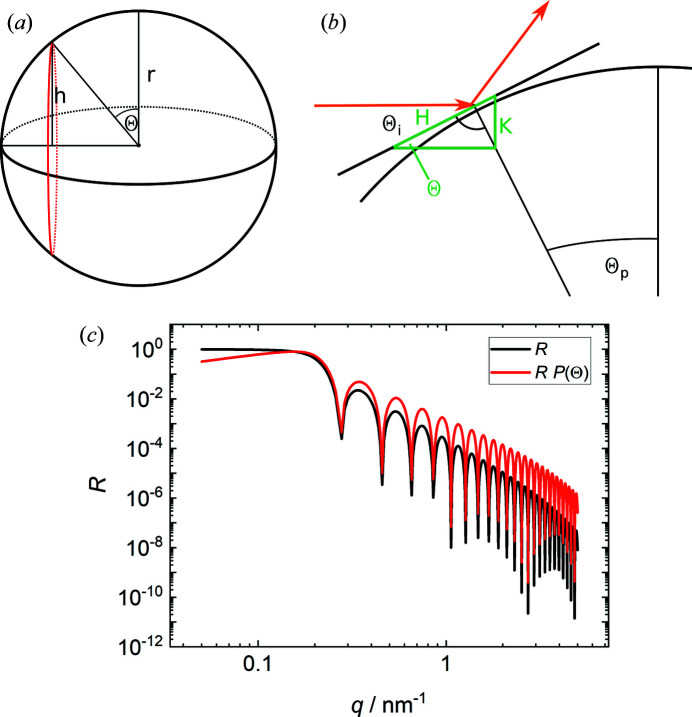
(*a*), (*b*) Illustrations of the angle correction, showing definitions of the various parameters used. (*c*) A comparison between an uncorrected reflectivity curve *R* of a planar layer (30 nm D_2_O layer in air, interfacial roughness σ = 0.3 nm, black line) and the same curve of a spherical cap taking the angle correction *R* *P*(Θ) (red line) into account. For better comparability with the uncorrected reflectivity curve, the maximum of the angle-corrected reflectivity curve is rescaled to 1.

Here, *h_i_* is the height corresponding to the surface section *i* and *r* is the radius of the sphere. Fig. 1 ▸(*a*) illustrates this for one specific value of Θ_i_. Note that Θ_i_ = Θ_p_ = Θ. With the relation *h* = *r*cos(Θ_p_) with the polar angle Θ_p_, *U*(Θ_i_) is given by

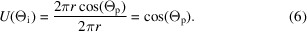

The probability of an angle of incidence Θ_i_ is given by the projection of a parallel beam onto a circle. This projection is illustrated in Fig. 1 ▸(*b*). 

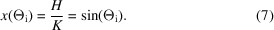

The combination of equations (6) and (7) yields the correction term *P*(Θ), which describes the distribution of angles of incidence for the reflection of a parallel beam from a sphere. 

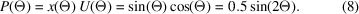

With the relation Θ = arcsin(*q*λ/4π), *P*(Θ) is given by

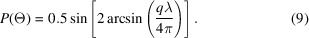

Including both correction factors *w_i_* and *P*(Θ), the scattered intensity *I* is 

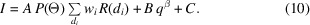

Here, *A* and *B* are scaling factors for the reflectivity and SAS contributions, respectively. *R*(*d_i_*) is an individual reflectivity curve for a D_2_O layer in air of thickness *d_i_*, β is the exponent of the SAS decay and *C* is the constant incoherent background.

The model in the form given in equation (10) is not capable of describing additional scattering features arising from aggregates or structures inside the foam films or plateau borders like micelles, as has already been reported in the literature (Axelos & Boué, 2003 ▸; Fameau *et al.*, 2011 ▸). It is possible to neglect the presence of micelles inside the foam films because the surfactant concentration is set to its CMC. Depending on the study and technique used, the CMC of C_14_TAB is reported to be between 3.4 and 3.6 m*M* (Mysels, 1955 ▸; Venable & Nauman, 1964 ▸; Simister *et al.*, 1992 ▸). Upon foaming, the surfactant molecules are gradually extracted from the foaming solution, leading to a reduced C_14_TAB concentration in the foam and consequently to the absence of micelles in the foam. This is beneficial for the development of a model for SANS from foams as it allows us to focus on the scattering features arising from the foam structure rather than the scattering from objects inside the foam film.

The fitting routine first determines *C* by averaging the 15 data points with the highest *q* values. Afterwards, *A*, *B*, β, *d*_0_ and σ are fitted to the data using the Nelder–Mead algorithm (Nelder & Mead, 1965 ▸). The individual reflectivity curves are calculated for a free-standing D_2_O layer in air using the matrix method (Daillant & Gibaud, 2009 ▸). The roughness of all air/water interfaces was fixed to the literature value of 0.3 nm (Braslau *et al.*, 1985 ▸).

**Figure 2:**
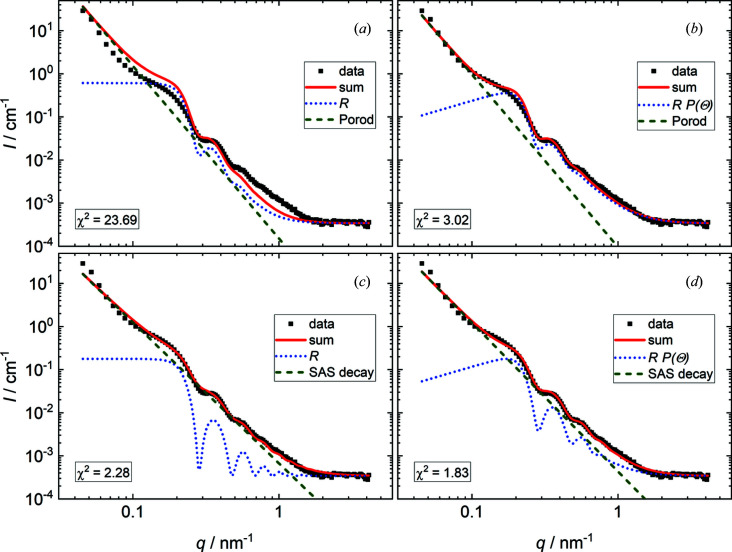
Comparisons of the best model fits with different assumptions. (*a*) Strict Porod decay without angle correction. (*b*) Strict Porod decay with angle correction. (*c*) SAS decay without angle correction. (*d*) SAS decay with angle correction. Experimental scattering data recorded at the lowest foam height (*h* = 7 cm, black squares) are shown together with the (angle-corrected) reflectivity (dotted blue lines), the Porod/SAS contribution (dashed green lines) and the total model fitting curve (solid red lines). The quality of the fit is judged by the value of χ^2^.

The model used in this article employs several assumptions that have not been made before now in models describing SANS data from foams: the introduction of polydispersity in the foam film thickness, the angle correction for the reflectivity term and the deviation from a strict Porod-type SAS contribution. The validity of the last two was checked by performing model fits without them. Fig. 2 ▸ shows the best model fits for the lowest foam height (*h* = 7 cm) for a series of models with different assumptions and the corresponding values of χ^2^. The experimental data (black squares) are shown together with the (angle-corrected) reflectivity (dotted blue lines), the Porod/SAS contribution (dashed green lines) and the total model fitting curve (solid red lines). In Figs. 2 ▸(*a*) and 2 ▸(*b*), a strict Porod-type SAS contribution (*B* *q* ^−4^) was used without and with the angle-corrected reflectivity term, respectively. In Figs. 2 ▸(*c*) and 2 ▸(*d*), a flexible SAS contribution (*B* *q* ^β^) was used without and with the angle-corrected reflectivity term, respectively. Both the angle correction and the use of a flexible SAS decay reduce χ^2^ by nearly one order of magnitude compared with the combination of a strict Porod decay and an uncorrected reflectivity term. Combining both assumptions further reduces χ^2^ by factors of 1.25 and 1.65, respectively, which suggests a certain validity and necessity of the assumptions to describe the presented scattering curve.

## 3. Results and discussion

### 3.1. SANS experiments and model fits

**Figure 3:**
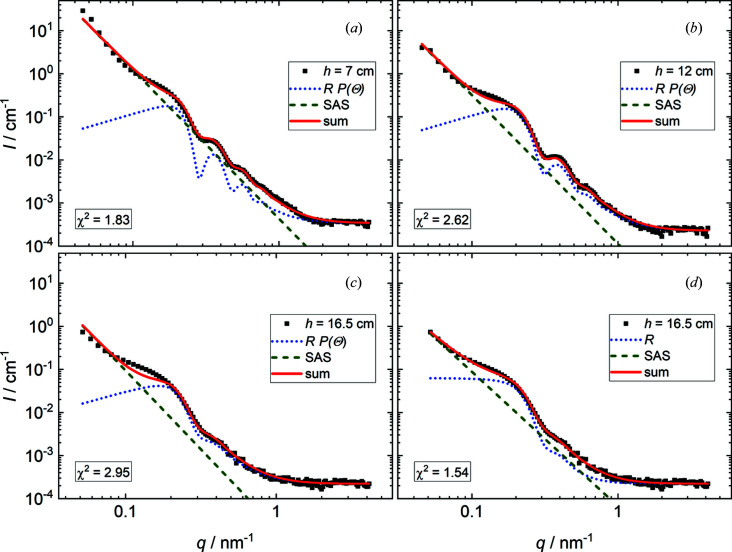
SANS data for a foam prepared from a C_14_TAB solution (*c* = 3.5 m*M* in D_2_O), measured at different foam heights. (*a*) *h* = 7 cm, (*b*) *h* = 12 cm, and (*c*), (*d*) *h* = 16.5 cm. Black squares are experimental data. Lines are model fits according to equation (10). For clarity, the full curves (solid red lines) are shown together with the SAS decay (dashed green lines) and the reflectivity contribution (dotted blue lines). In panels (*a*)–(*c*) the angle-corrected reflectivity *R* *P*(Θ) is applied. Panel (*d*) was modelled without the angle correction.

Fig. 3 ▸ shows SANS data for a foam prepared from a C_14_TAB solution (*c* = 3.5 m*M* in D_2_O), measured at different foam heights *h*, together with the model fits obtained using equation (10). In Figs. 3 ▸(*a*)–3 ▸(*c*) the angle-corrected reflectivity *RP*(Θ) was used to model the data. Fig. 3 ▸(*d*) was modelled without the angle correction. For the two lower foam heights, *h* = 7 and 12 cm [Figs. 3 ▸(*a*) and 3 ▸(*b*)], the angle-corrected model fits are in good agreement with the experimental data. For *h* = 16.5 cm, the angle-corrected model deviates significantly from the data at *q* ≃ 0.13 nm^−1^ [Fig. 3 ▸(*c*)]. Here, a description of the data with a reflectivity term without angle correction reduces χ^2^ by nearly a factor of 2 [Fig. 3 ▸(*d*)].

**Figure 4:**
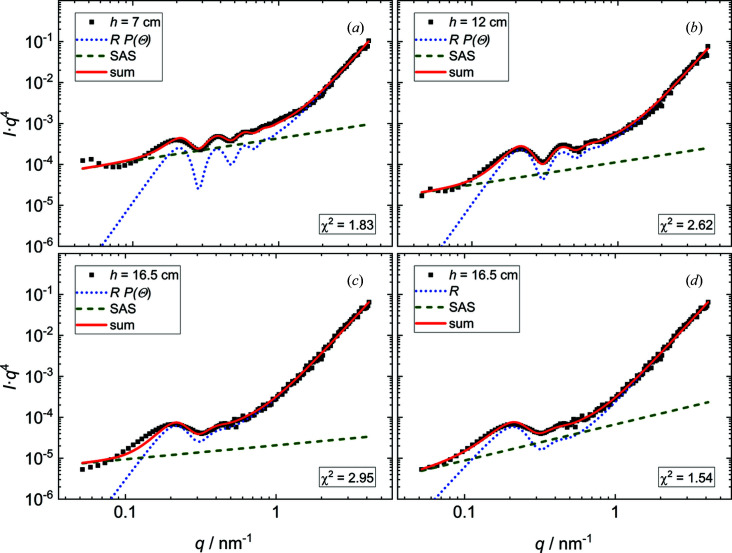
*I* versus *q* ^4^ plots of the data and model fits presented in Fig. 3 ▸. (*a*) *h* = 7 cm, (*b*) *h* = 12 cm, and (*c*), (*d*) *h* = 16.5 cm. Black squares are experimental data. Lines are model fits according to equation (10). For clarity, the full curves (solid red lines) are shown together with the SAS decay (dashed green lines) and the reflectivity contribution (dotted blue lines). In panels (*a*)–(*c*) the angle-corrected reflectivity *R* *P*(Θ) is applied. Panel (*d*) was modelled without the angle correction.

Since the overall decay in intensity is close to *I* ∝ *q* ^−4^, the data and model fits from Fig. 3 ▸ are presented in Fig. 4 ▸ in the Porod representation *I* *q* ^4^ versus *q*, which highlights the reflectivity features.

The fitted parameters of the best model fits for the different foam heights are summarized in Table 1 ▸. The fitted parameters are reflectivity scale factor *A*, mean foam film thickness *d*_0_, standard deviation of the foam film thickness σ, SAS scale factor *B*, power of the SAS decay β and incoherent background *C*.

With increasing foam height the overall scattered intensity decreases. This was also observed in various other reports investigating foams with SANS and is explained by drainage of the foam (Axelos & Boué, 2003 ▸; Ropers *et al.*, 2008 ▸; Micheau *et al.*, 2013 ▸; Yada *et al.*, 2020 ▸). During drainage the liquid films inside the foam become thinner and eventually rupture (Weaire & Hutzler, 1999 ▸; Kruglyakov *et al.*, 2008 ▸; Rio *et al.*, 2014 ▸). Consequently, the number of contrast-bearing objects in the foam decreases, which leads to a decrease in the scattered intensity.

This drainage process also explains the evolution of some of the SANS model fitting parameters with increasing foam height. The mean film thickness *d*_0_ decreases because of drainage. The reflectivity scale factor *A* decreases because of film rupturing. This process reduces the number of foam films in the sample cell, and consequently the probability of a reflectivity event occurring also decreases. Both drainage and rupturing of foam films lead to a reduction in the scattering volume inside the sample, and hence the incoherent background *C* decreases with increasing foam height.

The standard deviation of the foam film thickness σ increases with increasing foam height. This means that the thickness distribution of liquid films used in the model fit is broader, *i.e.* the film thickness becomes more polydisperse. Considering again the ageing processes described above, the increase in σ could be rationalized as follows. With proceeding rupturing of the foam films, the statistics of the film thickness distribution become worse and more susceptible to local thickness deviations. These thickness deviations might become more likely with increasing foam age as the liquid released during film breakup is incorporated by the remaining films, leading to a broader thickness distribution.

**Figure 5:**
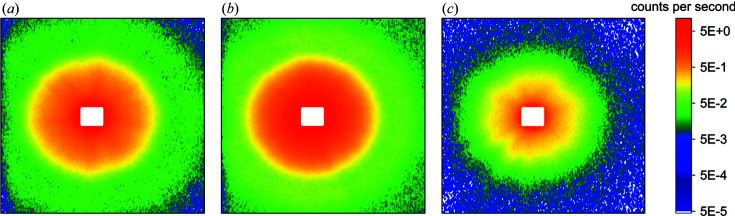
2D detector images of the SANS data measured at different foam heights. (*a*) *h* = 7 cm, (*b*) *h* = 12 cm, (*c*) *h* = 16.5 cm.

The SAS contribution to the overall signal can be interpreted as scattering from foam films under angles where reflection can be neglected, and from other structural motifs of the foam such as plateau borders and objects inside the foam (*e.g.* micelles or polymer chains) (Mikhailovskaya *et al.*, 2017 ▸). Previous publications on this topic mostly interpreted this contribution in terms of Porod’s law (Axelos & Boué, 2003 ▸; Schmidt *et al.*, 2010 ▸; Micheau *et al.*, 2013 ▸; Hurcom *et al.*, 2014 ▸; Mansour *et al.*, 2017 ▸, 2019 ▸; Yada *et al.*, 2020 ▸; Perticaroli *et al.*, 2020 ▸; Hohenschutz *et al.*, 2021 ▸), which describes the scattering from randomly oriented sharp interfaces and is characterized by a *q* ^−4^ power law regarding the decrease in the scattered intensity (Porod, 1951 ▸). However, in most of the studies cited above the decrease in scattered intensity over *q* was weaker than *q* ^−4^ (Axelos & Boué, 2003 ▸; Hurcom *et al.*, 2014 ▸; Micheau *et al.*, 2013 ▸; Yada *et al.*, 2020 ▸; Perticaroli *et al.*, 2020 ▸; Hohenschutz *et al.*, 2021 ▸). Therefore, the model used in this article also included β as a free parameter. β changes from −3.45 for both *h* = 7 and 12 cm to −3.12 for *h* = 16.5 cm (see Table 1 ▸). Scattering exponents between −3 and −4 can be interpreted either as scattering from surface fractals with a fractal dimension of *D*_S_ = 6 + β (Teixeira, 1988 ▸; Gommes *et al.*, 2021 ▸) or as a combination of multiple scattering power laws. An interpretation in terms of surface fractals would mean that the fractal dimension of the internal foam surface increases with increasing foam height. This would mean that the air/liquid interfaces inside the foam are not perfectly flat (*D*_S_ = 2) but have features normal to the interface, which can be interpreted as some type of roughness. The fact that this power law is present in the data even at very low *q* values would, however, imply a roughness of the order of several tens of nanometres, which is much too high for an air/liquid interface and therefore makes this interpretation not applicable. Note that the radius of curvature of foam bubbles in wet foams (*r*_B_ ≃ 0.1–1 mm) is too large to be detected by SANS measurements. Another interpretation is the combination of different SAS power laws. Here, the nodes and plateau borders would yield classic Porod scattering with β = −4, and the thinner plane-parallel portions of the films might be seen as randomly oriented flat objects, resulting in a SAS exponent of β = −2 (Gommes *et al.*, 2021 ▸). Upon drainage, the distribution of these scattering objects shifts towards the thin plane-parallel films and β increases. This argument is in line with the well known transition from spherical bubbles in wet foams with higher liquid volume fractions to polyhedron-shaped bubbles in dry foams with very low liquid volume fractions (Rio *et al.*, 2014 ▸; Drenckhan & Hutzler, 2015 ▸). This transition has already been observed in SANS experiments and associated with the appearance of ‘spikes’ in the 2D detector images (Axelos & Boué, 2003 ▸; Micheau *et al.*, 2013 ▸). This trend is also visible in the SANS data presented in this article. Fig. 5 ▸ shows 2D detector images of the SANS measurements at the three different foam heights. With increasing foam height the degree of radial symmetry in the scattering data decreases. At the highest measurement position several ‘spikes’ are visible, which are due to the decreased statistics regarding the orientation of the foam films because of coalescence.

Despite the fact that a transition from spherical to polyhedral bubbles can be explained by drainage and partly reflects the different structures inside a foam, the limited quantity of data presented here does not suffice to give a definite explanation for the observed change in β and therefore the nature of the SAS contribution to the overall scattering. Consequently, the SAS scaling factor *B* is not discussed further here. In order to improve the understanding of this contribution to the overall scattering signal, more detailed investigations, for example with an improved height resolution along the foam, are required.

The transition towards large polyhedral bubbles also explains the improved description of the SANS data without the angle-correction term at the highest measurement position in the foam. It can be assumed that here the foam bubbles are larger than at the lower measurement positions, which leads to a reduced number of foam films in the neutron beam. It appears that, in this situation, the number of foam films inside the beam is too small to justify the assumption that their orientation can be approximated by the projection of the incident beam onto a sphere. Here, the experimental data are better described with an even distribution of the angles of incidence. This finding supports the initial assumption that the bubble shape and the resulting orientational distribution of the foam films are relevant for the profile of the scattering curves. In addition, a rough estimate of the bubble shape can be made based on the SANS data from a foam.

All model fits used a constant SLD contrast between air (ρ_air_ = 0) and D_2_O (ρ_D2O_ = 6.34 × 10^−6^ Å^−2^). This can be rationalized as follows. As stated above, there are no micelles present in the foam films, because the concentration of C_14_TAB was set to its CMC. Considering this, the C_14_TAB molecules should mainly be located at the air/D_2_O interface, which results in D_2_O films with a very low concentration of surfactant molecules inside. Consequently, scattering from micelles in the foam was neglected. At concentrations above the CMC this assumption might not be valid anymore and scattering from micelles as well as a changing SLD will have to be considered.

The same approximation of only considering the air/D_2_O contrast was also made by previous studies investigating surfactant-stabilized foams using SANS (Ropers *et al.*, 2008 ▸; Micheau *et al.*, 2013 ▸; Hohenschutz *et al.*, 2021 ▸; Hurcom *et al.*, 2014 ▸). In the model presented in this article, the reflectivity contribution (dotted blue lines in Fig. 3 ▸) solely governs the oscillations in the scattering curves. Following this reflectivity-based interpretation, the first maximum in the scattering curves can be interpreted as a ‘pseudo-critical edge’ and its position is therefore only governed by the neutron contrast between the liquid films and the gas bubbles, and not by the film thickness, as already stated by Ropers *et al.* (2008 ▸) and Schmidt *et al.* (2010 ▸). All first maxima can be described by using an air/D_2_O contrast, which further supports the assumption that this contrast suffices to describe the scattering curves.

### 3.2. Comparison of SANS results with TFPB experiments

**Figure 6:**
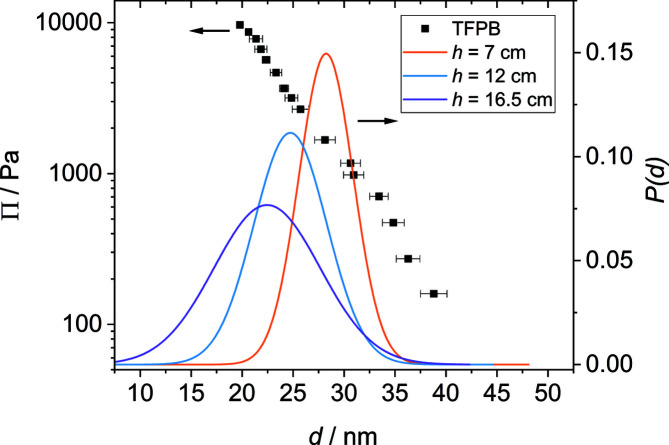
A comparison between foam film thicknesses extracted from SANS [*h* = 7 cm (orange), *h* = 12 cm (blue) and *h* = 16.5 cm (magenta), right *y* axis] and TFPB measurements (black squares, left *y* axis) with a 3.5 m*M* C_14_TAB solution.

In order to verify that the oscillations in the SANS curves can be attributed to the thickness of individual foam films, the disjoining pressure isotherm of an individual foam film was recorded with a TFPB. Fig. 6 ▸ shows a comparison between the Π versus *d* curve of an individual horizontal foam film, measured with a TFPB, and the film thickness distributions extracted from the model fitting parameters *d*_0_ and σ for the three different foam heights.

**Figure 7:**
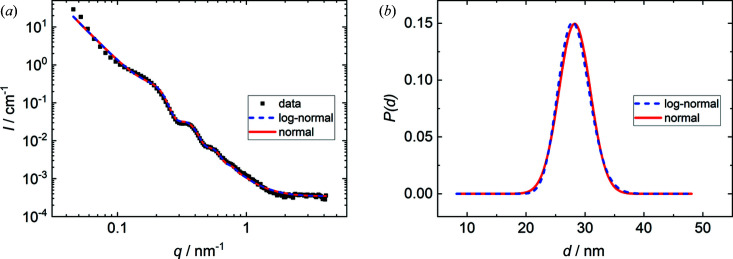
Comparison between normal and log-normal distributions of the foam film thicknesses in the fitting model. (*a*) Experimental scattering curve (black squares) and final model fits with normal (solid red line) and log-normal (dashed blue line) distributions, respectively. (*b*) Foam film thickness distributions of the model fits in (*a*).

The disjoining pressure Π is the pressure inside the foam film required to balance the external pressure in the measurement cell (Stubenrauch & Klitzing, 2003 ▸). Upon increasing the external pressure, the foam film becomes thinner and water is pushed back into the porous glass disc holding the film. The thickness of the individual foam film decreases from *d* = 46 nm at Π = 70 Pa to *d* = 19 nm at Π = 11 000 Pa. This is in good agreement with previous studies investigating the same system (Schulze-Schlarmann *et al.*, 2006 ▸; Bergeron, 1997 ▸). Note that the foam film inside the TFPB did not rupture at the highest pressure. Here, the upper limit of the instrumentally accessible pressure range was reached. The film thickness distributions extracted from the model fits lie mostly within the film thickness range measured with the TFPB and indicate that the disjoining pressure Π in films inside the foam ranges from ∼800 Pa to more than 11 000 Pa. The foam film thicknesses extracted from the model fits are in agreement with the results of the TFPB experiment (see Fig. 6 ▸), which validates the interpretation of the oscillations in the SANS curves in terms of foam film thicknesses. However, the film thickness distributions for *h* = 12 cm and *h* = 16.5 cm include film thicknesses thinner than the range covered by the TFPB measurement. This can be explained by the fact that the maximum experimentally accessible pressure was reached before the films ruptured. In addition, the films in a foam are more dynamic and do not adopt an equilibrium thickness as in a TFPB experiment. The thinnest films observed by SANS might also undergo a final non-equilibrium thinning step before rupturing that is not resolvable in a TFPB. A critical point in this context might also be an oversimplification of the thickness distribution of the foam films. This possibility was further investigated by changing the film thickness distribution in the model fit from a normal to a log-normal distribution. A comparison of the resulting model fits and the film thickness distributions for the lowest foam height (*h* = 7 cm) is shown in Fig. 7 ▸.

The final model fits and the resulting film thickness distributions are virtually identical. The mean thicknesses *d*_0_ are 28.26 and 28.24 nm for the fits with log-normal and normal distributions, respectively. The standard deviations of the thickness distribution σ are 2.68 and 2.67 nm. This means that an asymmetric distribution of the foam film thickness does not improve the agreement between experimental data and theoretical scattering curve and underlines that a normal distribution of the foam film thicknesses is a reasonable assumption.

## 4. Conclusion

Aqueous foams stabilized by the standard cationic surfactant tetradecyltrimethylammonium bromide (C_14_TAB) were studied using SANS and complementary TFPB experiments. We have developed a new model for the interpretation of SANS experiments on foams, which employs an incoherent superposition of weighted reflectivity curves and a SAS contribution. The reflectivity contribution is modified by an angle correction, which takes the distribution of angles of incidence on a spherical bubble into account. This model fully describes the scattering curves of surfactant-stabilized foams, yielding information about the thickness distribution of films inside the foam. With increasing foam height (corresponding to the time passed after foam formation) the mean film thickness decreases from 28 to 22 nm because of drainage. Simultaneously, the film thickness becomes more polydisperse. This is probably caused by film rupturing during coalescence and subsequent uptake of the released liquid by the remaining films.

The angle-corrected reflectivity contribution is not suitable for the description of the scattering curve of the driest foam (highest measurement position). Here, an uncorrected reflectivity term was sufficient to describe the experimental data, which is attributed to the transition from curved to faceted interfaces between bubbles in a polyhedral foam. In addition, the power law of the SAS contribution changes from β = −3.45 for wet foams to β = −3.12 for the driest foam. This is attributed to the progressive drainage and decomposition of larger structures in the foam-like plateau borders (β = −4) and the continuous emergence of plane-parallel films (β = −2) during foam ageing, and is again a hint towards a more polyhedral-like foam structure. The film thicknesses extracted from the model fits are comparable to the thicknesses of individual foam films in a TFPB experiment, further validating our model.

In summary, this paper presents a new model for the description of scattering data from foams, which takes into account reflectivity and small-angle scattering as well as the polydispersity of the foam films. Since the angle-corrected reflectivity term proposed in this paper is only suitable for the description of wet foams with curved bubble interfaces, a future challenge in this field will be a more realistic model for the reflectivity contribution in dry polyhedral foams.

## Supplementary Material

Small-angle neutron scattering data recorded at the D33 intrument at the Institut Laue-Langevin.: https://doi.org/10.5291/ILL-DATA.9-12-600

## Figures and Tables

**Table 1:** Fitting parameters of the model fits employing equation (10) for the three different foam heights The corresponding curves are shown in Figs. 3 ▸(*a*), 3 ▸(*b*) and 3 ▸(*d*).

*h* (cm)	*A* (cm^−1^)	*d*_0_ (nm)	σ (nm)	*B* × 10^−8^	β	*C* × 10^−4^ (cm^−1^)
7	0.18	28.2	2.7	15.49	−3.45	3.43
12	0.17	24.7	3.6	4.09	−3.45	2.30
16.5	0.06	22.4	5.2	5.09	−3.12	2.22
